# Structural Connectivity Disruption and Structural–Functional Decoupling in Working Memory Networks Across Pre‐Dialysis and Maintenance Hemodialysis End‐Stage Renal Disease Patients

**DOI:** 10.1002/cns.70761

**Published:** 2026-01-19

**Authors:** Xiaoling Xu, Shaohui Ma, Siyao Liu, Zhaoyao Luo, Qiange Zhu, Huijie Yuan, Xinyi Zhu, Wen Gu, Peng Li, Jianjun Zhang, Ming Zhang, Junya Mu

**Affiliations:** ^1^ Department of Medical Imaging First Affiliated Hospital of Xi'an Jiaotong University Xi'an the People's Republic of China; ^2^ Department of Radiology & Functional and Molecular Imaging Key Lab of Shaanxi Province Tangdu Hospital, Fourth Military Medical University Xi'an the People's Republic of China; ^3^ Department of Radiology Zhejiang Hospital Hangzhou the People's Republic of China

**Keywords:** end‐stage renal disease, hemodialysis, structural‐functional coupling, working memory

## Abstract

**Aims:**

End‐stage renal disease (ESRD) is associated with working memory (WM) impairment. We assessed how structural connectivity (SC), functional connectivity (FC), and structural–functional coupling (SFC) differ between pre‐dialysis ESRD (ESRDp), maintenance hemodialysis ESRD (ESRDm), and healthy controls (HCs), and how these changes relate to serum markers and WM performance.

**Methods:**

29 ESRDp, 29 ESRDm, and 46 HCs completed 0‐, 1‐, and 2‐back tasks, diffusion MRI, and fMRI. WM nodes were defined by overlaying a meta‐analytic map with the Harvard–Oxford atlas. SC, FC, and regional SFC were computed among WM‐related regions. Group differences, correlations with serum markers, and mediation models were examined.

**Results:**

ESRDp showed markedly lower n‐back accuracy, longer reaction time (RT), reduced frontoparietal SC, and widespread SFC reductions compared with ESRDm and HCs, whereas ESRDm exhibited near‐normal WM performance and partially restored SC/SFC. Elevated urea and lower sodium in ESRDp were associated with weaker SC and altered SFC, which related to poorer accuracy and slower RT; SC and SFC significantly mediated these associations.

**Conclusion:**

ESRDp is characterized by disruption and decoupling of WM networks, while ESRDm is associated with partial network normalization. WM‐related SC and SFC combined with serum markers may help identify cognitive vulnerability in ESRD.

**Trial Registration:**

ClinicalTrials.gov identifier: NCT03961724

## Graphical abstract

This study examined structural connectivity (SC) and structural–functional coupling (SFC) within working memory (WM) networks in end‐stage renal disease. Pre‐dialysis patients showed disrupted frontoparietal connectivity and reduced coupling, associated with higher urea, lower sodium, and impaired WM performance. Maintenance hemodialysis patients exhibited near‐normal WM and partially restored network organization, suggesting WM‐related SC and SFC as indicators of cognitive vulnerability.

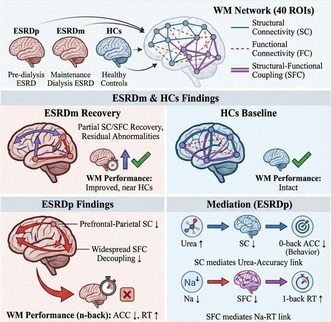

**Abbreviations**
ACC: accuracy
Angular_L: left angular gyrus
ANOVA: analysis of variance
BBB: blood–brain barrier
Caudate_L: left caudate
CBF: cerebral blood flow
DTI: diffusion tensor imaging
ESRD: end‐stage renal disease
ESRDm: maintenance hemodialysis end‐stage renal disease patients
ESRDp: pre‐dialysis end‐stage renal disease patients
F_Ope_L: left frontal operculum cortex
FC: functional connectivity
FDR: false discovery rate
FP_L: left frontal pole
FP_R: right frontal pole
HCs: healthy controls
IFG_PO_L: left inferior frontal gyrus, pars opercularis
IFG_PT_L: left inferior frontal gyrus, pars triangularis
Insular_L: left insular cortex
JLC_R: right juxtapositional lobule cortex
LOC_SD_L: left lateral occipital cortex, superior division
MFG_R: right middle frontal gyrus
Post_L: left postcentral gyrus
Pre_L: left precentral gyrus
ROI: region of interest
rs‐fMRI: resting‐state functional magnetic resonance imaging
RT: reaction time
SC: structural connectivity
SFC: structural‐functional coupling
SFG_L: left superior frontal gyrus
SFG_R: right superior frontal gyrus
SG_AD_L: left supramarginal gyrus, anterior division
WM: working memory

## 1 Background

End‐stage kidney disease (ESRD) is a common chronic condition characterized by a glomerular filtration rate of less than 15 mL/min/1.73 m^2^. Studies indicate that 30%–60% of ESRD patients experience varying degrees of cognitive dysfunction [1, 2, 3]. Working memory (WM) is a core component of higher‐order cognitive functions, and such deficits in ESRD patients significantly compromise decision‐making abilities, treatment adherence, and quality of life, while also correlating with increased risks of hospitalization and mortality [3, 4, 5, 6]. However, the neural mechanisms underlying WM impairment in ESRD remain incompletely understood.

Hemodialysis is the most widely used renal replacement therapy for ESRD and is essential for maintaining biochemical homeostasis in advanced kidney failure [7]. By intermittently removing uremic solutes and correcting electrolyte and volume disturbances, maintenance hemodialysis could, in principle, mitigate some forms of cognitive decline [8, 9]. Consistent with this, our previous studies suggest that a single dialysis session transiently enhances resting‐state functional connectivity (FC) in the default mode network [10], while long‐term maintenance hemodialysis is associated with functional reorganization of the frontal–parietal network [11]. At the same time, ESRD‐related factors—such as persistent uremic burden, anemia, vascular dysfunction, and the repeated hemodynamic shifts that occur during dialysis sessions—may continue to exert harmful effects on the brain [4, 12, 13, 14, 15, 16, 17, 18, 19, 20]. Neuroimaging evidence is in line with this complex picture, demonstrating reduced FC and SC within prefrontal–parietal networks in ESRD patients, with inverse correlations between connectivity strength and serum urea levels [18, 21, 22]. However, it remains unclear how WM‐related brain networks differ between patients who have not yet started dialysis and those on stable maintenance hemodialysis.

Brain dysfunction in ESRD has most often been characterized by looking at either structural or functional connectivity in isolation. Structural–functional coupling (SFC) has been introduced as an index of how closely a region's anatomical wiring supports its pattern of functional connections. SFC summarizes the correspondence between connection strengths in the structural network and the associated functional links for each node, providing a measure of local structure–function alignment [23, 24]. Reduced SFC is thought to indicate a mismatch between physical pathways and dynamic communication and has been associated with inefficient network organization and cognitive difficulties in several brain disorders [25, 26, 27]. However, SC, FC, and SFC within WM‐related networks have not yet been systematically examined together in ESRD, particularly in relation to dialysis status and routinely monitored biochemical disturbances.

In this study, we investigated SC, FC, and SFC within WM‐related networks across three groups: healthy controls (HCs), pre‐dialysis ESRD patients (ESRDp), and maintenance hemodialysis ESRD patients (ESRDm). We hypothesized that ESRDp would show more pronounced WM impairment and greater disruption and decoupling of WM‐related SC, FC, and SFC than ESRDm and HCs, and that ESRDm would exhibit intermediate, partially normalized patterns. We further examined how these connectivity alterations relate to routinely monitored serum biomarkers within ESRD and explored whether SC, FC, or SFC statistically mediate associations between selected biochemical indices and WM performance. By integrating behavioral, biochemical, and SC/FC/SFC, we aimed to clarify how dialysis status and systemic disturbances are associated with WM network in ESRD.

## 2 Methods

The protocol was evaluated and authorized by the Ethics Committee of the First Affiliated Hospital, School of Medicine, Xi'an Jiaotong University (approval ID XJTU1AF‐CRF‐2018‐006). All procedures adhered to the ethical principles of the Declaration of Helsinki. The study was prospectively listed in the ClinicalTrials.gov registry (NCT03961724). Before enrollment, every participant received a detailed explanation of the study procedures and then signed a written consent form.

### 2.1 Participants

We enrolled 29 ESRDp, 29 ESRDm, and 46 HCs between July 2019 and December 2025. The three groups were comparable in age, years of education, and sex distribution. All ESRD diagnoses were confirmed by kidney biopsy. Among the 58 ESRD patients, the primary renal pathologies included mesangiocapillary glomerulonephritis (ESRDp: 15; ESRDm: 10), endocapillary proliferative glomerulonephritis (ESRDp: 8; ESRDm: 12), sclerosing glomerulonephritis (ESRDp: 0; ESRDm: 2), focal segmental glomerulosclerosis (ESRDp: 4; ESRDm: 2), IgA nephropathy (ESRDp: 2; ESRDm: 2), and membranous nephropathy (ESRDp: 0; ESRDm: 1). All patients met criteria for ESRD with an estimated glomerular filtration rate < 15 mL/min/1.73 m^2^. ESRDm patients had been on thrice‐weekly hemodialysis for more than 12 months, whereas ESRDp patients were scheduled to initiate hemodialysis for the first time.

Exclusion criteria for all participants were: (a) macroscopic T2‐hyperintense brain lesions on MRI, regardless of size; (b) history of neurological disease; (c) significant physical disability that would preclude MRI or task performance; (d) current or past alcohol, nicotine, or illicit drug abuse; (e) diabetic nephropathy or primary hypertensive nephropathy as the cause of renal failure; and (f) claustrophobia or other contraindications to MRI.

### 2.2 Blood Biochemistry

For the ESRDp and ESRDm groups, venous blood samples were obtained within 3 days prior to MRI scanning. Routine biochemical assays included hemoglobin (Hb), parathyroid hormone (PTH), potassium (K), calcium (Ca), creatinine, urea, cystatin C, sodium (Na), and phosphorus (P). Healthy controls did not undergo blood biochemistry testing as part of this study.

### 2.3 WM Assessment: N‐Back Task

WM was evaluated using a standard numeric n‐back paradigm with three load levels (0‐, 1‐, and 2‐back). On each trial, a single numeral appeared at the center of the screen for 500 ms, followed by a 2500‐ms blank interval. In the 0‐back condition, participants pressed a button whenever a pre‐specified target digit was shown. In the 1‐back condition, they responded if the current digit was identical to the one presented on the immediately preceding trial, and in the 2‐back condition if it matched the digit from two trials earlier, thereby imposing higher working‐memory demands. Each level included a short practice run, and brief rest periods were offered between blocks. For every condition, we recorded response latency (RT; time from stimulus onset to keypress) and performance accuracy (ACC; percentage of correct responses).

### 2.4 Acquisition of MRI Data

A 3.0 Tesla GE Signa Excite system (GE Medical Systems, Milwaukee, WI, USA) with an eight‐channel head coil was used to acquire all MRI scans.

We employed a single‐shot echo‐planar sequence for diffusion tensor imaging (DTI). Diffusion weighting was implemented in 30 non‐collinear directions (*b* = 1000 s/mm^2^), along with five images devoid of diffusion weighting (b0). The sequence encompassed 75 contiguous axial slices (slice thickness 2 mm; field of view 256 mm; matrix 128 × 128; TR 9400 ms; TE 84 ms), yielding isotropic voxels of 2 mm^3^.

Resting‐state functional magnetic resonance imaging (rs‐fMRI) was obtained while participants rested quietly with their eyes closed, instructed to remain awake and refrain from intentional cognitive engagement. The parameters for rs‐fMRI acquisition were as follows: Thirty axial slices; repetition time (TR) = 2000 ms; echo time (TE) = 30 ms; flip angle = 90°; slice thickness = 5 mm; no gap; matrix = 64 × 64; field of view (FOV) = 240 × 240 mm^2^. Each run consisted of 205 volumes, resulting in a total scan duration of 410 s per participant.

### 2.5 Resting‐State fMRI Preprocessing

The preprocessing of resting‐state data was conducted using DPARSF (http://www.restfmri.net/forum/DPARSF). The initial 10 time points were eliminated to enable magnetization to attain a steady state. The residual volumes were adjusted for slice‐timing discrepancies and realigned to the initial image to compensate for head movement. Subsequently, images were spatially normalized to the MNI template utilizing the EPI reference image and resampled to a resolution of 3 × 3 × 3 mm^3^. A Gaussian kernel with a 6‐mm full width at half maximum was utilized for spatial smoothing. Linear drifts were eliminated, and a temporal band‐pass filter (0.01–0.08 Hz) was applied to preserve low‐frequency variations of significance. Nuisance regression was conducted on the normalized data to mitigate non‐neuronal noise, incorporating a 24‐parameter head‐motion model and mean signals from white‐matter and cerebrospinal fluid masks obtained from segmented T1‐weighted images in MNI space [28, 29].

### 2.6 DTI Preprocessing

DTI data were analyzed using ExploreDTI (www.exploredti.com) [30]. Preprocessing included correction for subject motion and eddy‐current/EPI distortions, skull stripping, and diffusion tensor fitting to generate fractional anisotropy (FA) maps. Deterministic whole‐brain fiber tracking was subsequently conducted in native space, with an FA threshold of 0.20 and a maximum permissible rotation angle of 30°. The resultant tractography became the foundation for the construction of the SC networks.

### 2.7 Definition of WM‐Related Nodes

WM–related regions were identified using a two‐step procedure that combined task‐based meta‐analysis with an anatomical atlas. First, we queried the Neurosynth platform (https://www.neurosynth.org/) using the term “working memory,” yielding 1091 relevant functional neuroimaging studies. A meta‐analytic Z‐map in MNI space was generated and thresholded with a cluster‐level cutoff of *z* > 4.5 and FDR‐corrected *p* < 0.01, producing a binarized WM mask reflecting regions consistently recruited by WM tasks. Second, this mask was overlaid with the 112‐region Harvard–Oxford cortical and subcortical atlas, which is also defined in MNI space and therefore allows direct voxelwise intersection. For each atlas parcel, we assessed the overlap with the WM mask; regions with at least 10 overlapping voxels were retained as WM‐related regions of interest (ROIs), whereas parcels with minimal or no overlap were excluded. ROIs were not merged across atlas boundaries, so each network node corresponded to a single Harvard–Oxford region. In total, 40 WM‐related ROIs were selected as nodes for subsequent functional and structural connectivity analyses. Anatomical labels and MNI coordinates are listed in Table S1.

### 2.8 Functional and Structural Network Construction

For each participant, FC and SC were calculated using the predefined WM‐related ROI nodes. Functional networks were derived by defining 40 ROIs as nodes, with edges representing interregional FC. The blood oxygen level‐dependent signal for each node was calculated by averaging preprocessed time series across all voxels within the ROI. Pairwise Pearson correlation coefficients between all 40 nodes were computed and Fisher z‐transformed to generate an FC matrix.

Structural networks were built using deterministic wholebrain white matter tractography in ExploreDTI to quantify SC between the same 40 nodes. Tractography used the tensor‐derived FA maps with a seed FA threshold of 0.2 and a maximum turning angle of 30 degrees. To reduce spurious streamlines, fibers shorter than 20 mm were excluded, and unweighted adjacency matrices were created by retaining only connections with ≥ 3 streamlines.

### 2.9 Calculation of Structural Functional Coupling

Regional SFC was quantified following previous work [23, 31, 32]. For each subject, we extracted the row of the SC matrix and the corresponding row of the FC matrix (excluding the diagonal) for each ROI. The Spearman rank correlation was computed between these two vectors, yielding a single SFC value per region. This procedure produced a 40‐element SFC vector for each participant, indexing the degree to which FC patterns align with the underlying SC profile at each WM‐related node.

### 2.10 Statistical Analysis

Statistical analysis was conducted using SPSS version 26.0 (IBM Corp., Armonk, NY, USA). The distribution of all continuous variables, encompassing demographic indices, blood biochemistry metrics, and n‐back accuracy and reaction time, was assessed using the Shapiro–Wilk test. The results of the normality tests for all datasets are reported in the results section of the Supporting Information. The group‐difference and correlation analyses were adjusted for multiple comparisons using the false discovery rate (FDR) procedure; values with *p*_FDR < 0.05 were considered statistically significant.

Differences in age and years of education across ESRDp, ESRDm, and HCs were analyzed with one‐way ANOVA or the Kruskal–Wallis test (depending on distribution). Post hoc contrasts were carried out using two‐samples *t*‐tests or Mann–Whitney *U* tests. Sex distributions were compared using *χ* ^2^ tests. Laboratory variables were contrasted between ESRDp and ESRDm using two‐samples *t*‐tests or Mann–Whitney *U* tests with FDR correction.

To evaluate WM performance, separate 3 (load: 0‐, 1‐, 2‐back) × 3 (group: ESRDp, ESRDm, HCs) mixed‐model repeated‐measures ANOVAs were conducted for ACC and RT. Significant main effects and interactions were followed by FDR‐corrected pairwise group comparisons (*t*‐tests or Mann–Whitney *U* tests, depending on distribution).

Between‐group differences in FC, SC, and SFC were assessed using one‐way ANOVA at each edge (for FC/SC) or node (for SFC), with post hoc independent‐samples *t*‐tests across all tested connections or regions. Pearson correlation analyses combined with 1000 permutation tests were carried out separately in ESRDp and ESRDm to examine relationships among altered FC, SC, and SFC metrics, n‐back performance, and blood biochemistry. Only variables that showed significant intergroup differences were included, and the resulting *p*‐values were FDR‐adjusted.

Finally, mediation analyses were implemented in SPSS using the PROCESS macro (version 3.4) with 5000 bootstrap resamples to test whether FC, SC, or SFC measures statistically mediated the associations between laboratory indices and WM performance in ESRDp and ESRDm. Given the established links of anemia and secondary hyperparathyroidism with cognitive function in ESRD, Hb and PTH were entered as covariates in all mediation models. Indirect effects with *p* < 0.05 were considered significant.

## 3 Results

### 3.1 Results of Demographic and Clinical Data and Working‐Memory Performance

Table 1 presents the demographic profile of the three groups. Kruskal–Wallis tests showed no between‐group differences in age or years of education for ESRDp, ESRDm, and HCs. Likewise, the proportion of males and females did not differ significantly across groups.

**TABLE 1:** Demographic and n‐back working memory variable of HCs, ESRD_p_ and ESRD_m_.

Variable	HCs	ESRD_p_	ESRD_m_	*H*/*F*/*χ* ^2^	*p*	*Z*_1_/*t*_1_	*p*_1_	*Z*_2_/*t*_2_	*p*_2_	*Z*_3_/*t*_3_	*p*_3_
	(*n* = 46)	(*n* = 29)	(*n* = 29)								
	Mean (SD)	Mean (SD)	Mean (SD)								
Age (years)	35.312 (2.101)	34.346 (1.706)	33.286 (1.463)	0944^a^	0.624						
Gender (M/F)	23/23	20/9	21/8	4.713^b^	0.095						
Education (years)	11.635 (0.511)	11.192 (0.746)	11.536 (0.523)	0.414^a^	0.813						
0‐back ACC	0.926 (0.011)	0.718 (0.058)	0.917 (0.027)	20.808^a^	< 0.001	4.000^c^	< 0.001	0.939^c^	0.347	−3.573^c^	< 0.001
0‐back RT	449.678 (9.144)	583.524 (19.025)	447.634 (24.963)	32.733^a^	< 0.001	−7.076^d^	< 0.001	1.714^c^	0.086	4.518^c^	< 0.001
1‐back ACC	0.814 (0.015)	0.577 (0.041)	0.811 (0.027)	29.325^a^	< 0.001	4.963^c^	< 0.001	0.445^c^	0.657	−4.381^c^	< 0.001
1‐back RT	524.436 (13.810)	674.028 (23.857)	523.669 (23.324)	24.639^a^	< 0.001	−5.828^d^	< 0.001	0.527^c^	0.598	4.155^c^	< 0.001
2‐back ACC	0.743 (0.021)	0.518 (0.043)	0.740 (0.035)	21.408^a^	< 0.001	4.458^c^	< 0.001	0.180^c^	0.857	−3.475^c^	< 0.001
2‐back RT	571.547 (14.500)	681.738 (22.118)	559.157 (25.415)	10.109^e^	< 0.001	−4.351^d^	< 0.001	−0.454^d^	0.651	3.615^d^	< 0.001

*Note: Z*1/*t*1/*p*1: Statistical comparison between HCs and ESRD_p_ groups; *Z*2/*t*2/*p*2: Statistical comparison between HCs and ESRD_m_ groups; *Z*3/*t*3/*p*3: Statistical comparison between ESRD_p_ and ESRD_m_ groups.

Abbreviations: ACC, accuracy; ESRD, end‐stage renal disease; ESRD_m_, maintenance hemodialysis ESRD patients; ESRD_p_, pre‐dialysis ESRD patients; HCs, Healthy Controls; RT, reaction time.

^a^ Kruskal–Wallis *H* test.

^b^ Chi‐square test.

^c^ Mann–Whitney *U* test.

^d^ Two‐sample *t*‐test.

^e^ ANOVA.

For ACC, both load (*F* (2,186) = 83.77, *p* < 0.001) and group (*F* (2,101) = 21.77, *p* < 0.001) showed significant main effects, whereas the load‐by‐group interaction was not significant (*F* (4,186) = 0.24, *p* = 0.92). For RT, we again observed robust main effects of load (*F* (2,186) = 109.52, *p* < 0.001) and group (*F* (2,101) = 18.19, *p* < 0.001), with no significant interaction between load and group (*F* (4,186) = 1.59, *p* = 0.18).

After applying FDR correction, post hoc comparisons indicated that ESRDp patients performed worst on the n‐back task. Across all loads (0‐, 1‐, and 2‐back), their ACC was lower than that of both ESRDm patients and HCs (all *p*_FDR < 0.001), whereas ACC did not differ between the ESRDm and HC groups (all *p*_FDR > 0.05; Table 1; Figure S1). RTs showed a similar pattern: at each load, ESRDp participants responded more slowly than ESRDm patients and HCs (all *p*_FDR < 0.001), while RTs were comparable between ESRDm and HCs (all *p*_FDR > 0.05; Table 1; Figure S1).

Blood biochemistry tests in ESRDp and ESRDm are summarized in Table 2 and Figure S2. When controlling for multiple comparisons across laboratory indices, ESRDp patients had higher urea (*t* = 2.567, *p*_FDR = 0.043), but lower Na (*z* = −3.784, *p*_FDR = 0.002) and PTH levels (*z* = −3.982, *p*_FDR < 0.001) than ESRDm patients. Calcium (*z* = −2.171, *p*_FDR = 0.069) and hemoglobin (*z* = −2.027, *p*_FDR = 0.078) tended to be lower in ESRDp but did not remain statistically significant after FDR correction. Other hematological parameters, including potassium (*t* = −1.482, *p*_FDR = 0.183), phosphate (*t* = 1.544, *p*_FDR = 0.124), and cystatin C (*z* = 0.260, *p*_FDR = 0.801), showed no significant differences between groups.

**TABLE 2:** Disease status and blood biochemistry tests between ESRD_p_ and ESRD_m_.

Variable	ESRD_p_	ESRD_m_	*Z*/*t*	*p*‐FDR
	(*n* = 29)	(*n* = 29)		
	Mean (SD)	Mean (SD)		
Dialysis time (months)	—	32.929 (4.810)		
Creatinine (μmol/L)	945.000 (79.284)	914.786 (47.234)	0.606^a^	0.802
Urea (μmol/L)	31.417 (2.369)	24.014 (1.435)	2.567^b^	0.043
Potassium (mmol/L)	4.745 (0.159)	4.975 (0.141)	−1.482^b^	0.183
Phosphate (mmol/L)	1.986 (0.098)	1.793 (0.079)	1.544^b^	0.124
Calcium (mmol/L)	1.914 (0.074)	2.129 (0.042)	−2.171^a^	0.069
Sodium (mmol/L)	140.800 (0.691)	144.000 (0.655)	−3.784^b^	0.002
Cystatin C (mg/L)	4.560 (0.234)	4.479 (0.246)	0.260^a^	0.801
Hemoglobin (g/L)	86.450 (4.581)	102.393 (3.824)	−2.027^a^	0.078
Parathyroid hormone (pg/mL)	339.950 (39.442)	742.929 (85.157)	−3.982^a^	< 0.001

Abbreviations: ESRD_m_, maintenance hemodialysis end‐stage renal disease patients; ESRD_p_, pre‐dialysis end‐stage renal disease patients.

^a^ Mann–Whitney *U* test.

^b^ Two‐sample *t*‐test.

### 3.2 Intergroup Comparison of Working Memory Related SC, FC, and SFC

**FIGURE 1:**
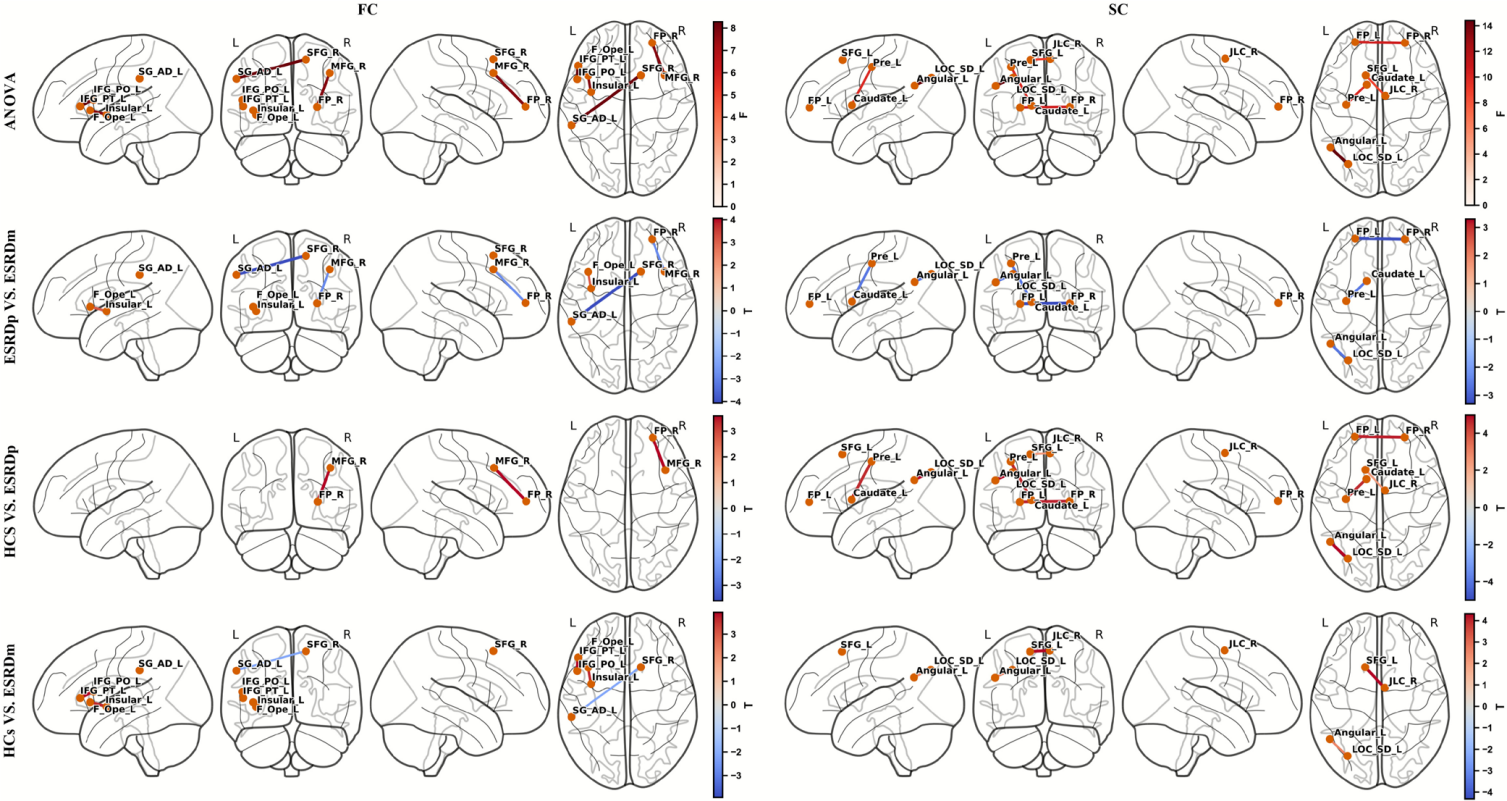
Group differences in FC and SC among ESRDp, ESRDm, and HCs. Left and right columns show FC and SC results, respectively. The first row displays edges with significant main effects of group from one‐way ANOVA across ESRDp, ESRDm, and HCs. The second to fourth rows show post hoc pairwise comparisons (ESRDp vs. ESRDm, HCs vs. ESRDp, and HCs vs. ESRDm, respectively). In the ANOVA panels, edge colors indicate the presence and magnitude of a group effect (no directionality). In the pairwise comparison panels, red edges denote significantly higher connectivity and blue edges denote significantly lower connectivity in the group listed first. All displayed connections survive FDR correction for multiple comparisons. Angular_L, left angular gyrus; Caudate_L, left caudate; ESRDm, maintenance hemodialysis end‐stage renal disease patients; ESRDp, pre‐dialysis end‐stage renal disease patients; F_Ope_L, left frontal operculum cortex; FC, functional connectivity; FP_L, left frontal pole; FP_R, right frontal pole; HCs, healthy controls; IFG_PO_L, left inferior frontal gyrus, pars opercularis; IFG_PT_L, left inferior frontal gyrus, pars triangularis; Insular_L, left insular cortex; JLC_R, right juxtapositional lobule cortex (formerly supplementary motor cortex); LOC_SD_L, left lateral occipital cortex, superior division; MFG_R, right middle frontal gyrus; Pre_L, left precentral gyrus; SC, structural connectivity; SFG_L, left superior frontal gyrus; SFG_R, right superior frontal gyrus; SG_AD_L, left supramarginal gyrus, anterior division.

As shown in Figure 1, after FDR correction, one‐way ANOVA on SC revealed significant group differences in FP_L–FP_R, Angular_L–LOC_SD_L, SFG_L–JLC_R, and Pre_L–Caudate_L. Post hoc analyses demonstrated that, compared with HCs, ESRDp patients exhibited significantly reduced SC across all four connections. Relative to ESRDp, ESRDm patients showed partial SC recovery in FP_L–FP_R, Angular_L–LOC_SD_L, and Pre_L–Caudate_L. However, SC in Angular_L–LOC_SD_L and SFG_L–JLC_R remained lower in ESRDm than in HCs, indicating persistent structural disruption in these pathways.

As also shown in Figure 1, after FDR correction, ANOVA on FC identified significant group differences in FP_R–MFG_R, IFG_PT_L–IFG_PO_L, SFG_R–SG_AD_L, and Insular_L–F_Ope_L. Relative to HCs, ESRDp patients exhibited significantly reduced FC in FP_R–MFG_R. ESRDm patients, compared with HCs, showed decreased FC in IFG_PT_L–IFG_PO_L and Insular_L–F_Ope_L but increased FC in SFG_R–SG_AD_L. Compared with ESRDp, ESRDm patients showed significant restoration of FC in FP_R–MFG_R and SFG_R–SG_AD_L, whereas FC in Insular_L–F_Ope_L further deteriorated.

**FIGURE 2:**
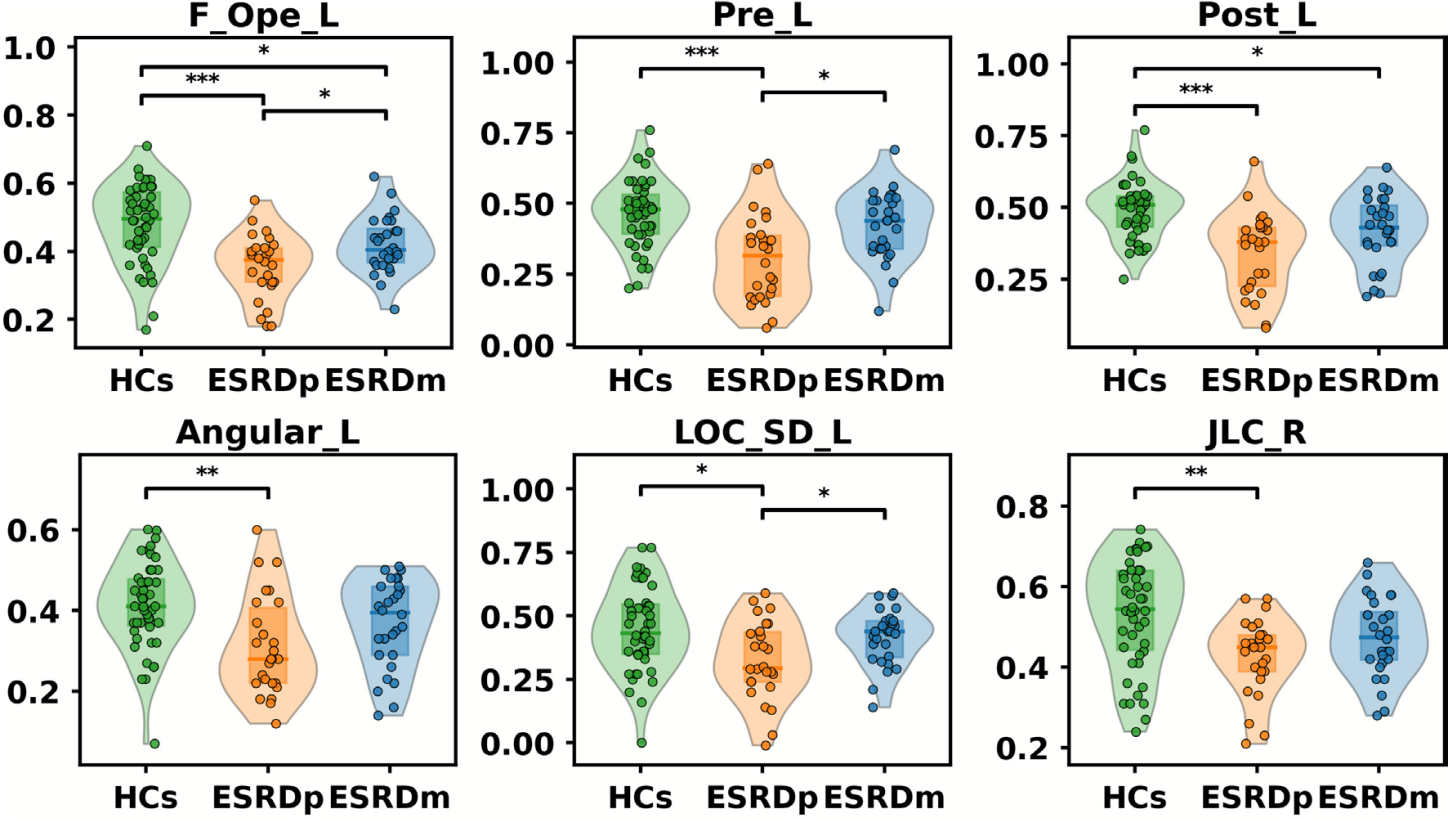
Group differences in SFCin regions showing a significant difference of ANOVA. Compared with HCs, ESRDp exhibited reduced SFC in all six regions, whereas ESRDm showed reduced SFC relative to HCs in Post_L, JLC_R, and F_Ope_L. ESRDp further showed lower SFC than ESRDm in Pre_L, LOC_SD_L, and F_Ope_L. Violin plots show the distribution of SFC values for HCs, ESRDp, and ESRDm; dots represent individual participants. Asterisks denote comparisons that remain significant after FDR correction (*p*_FDR < 0.05, **p_FDR < 0.01, ***p_FDR < 0.001). Angular_L, left angular gyrus; ESRDm, maintenance hemodialysis end‐stage renal disease patients; ESRDp, pre‐dialysis end‐stage renal disease patients; F_Ope_L, left frontal operculum cortex; HCs, healthy controls; JLC_R, right juxtapositional lobule cortex (formerly supplementary motor cortex); LOC_SD_L, left lateral occipital cortex, superior division; Post_L, left postcentral gyrus; Pre_L, left precentral gyrus; SFC, structural–functional coupling.

As shown in Figure 2 and Table S2, one‐way ANOVA identified significant group differences in SFC across six regions: Pre_L, JLC_R, F_Ope_L, Angular_L, LOC_SD_L, and Post_L. Relative to HCs, ESRDp exhibited widespread SFC reductions across all six regions. ESRDm showed significant SFC reductions in Post_L and F_Ope_L relative to HCs. Compared with ESRDp, ESRDm patients exhibited significantly higher SFC in Pre_L, LOC_SD_L, and F_Ope_L.

### 3.3 Correlation Analysis of Clinical Blood Indicators and Working Memory Load

**FIGURE 3:**
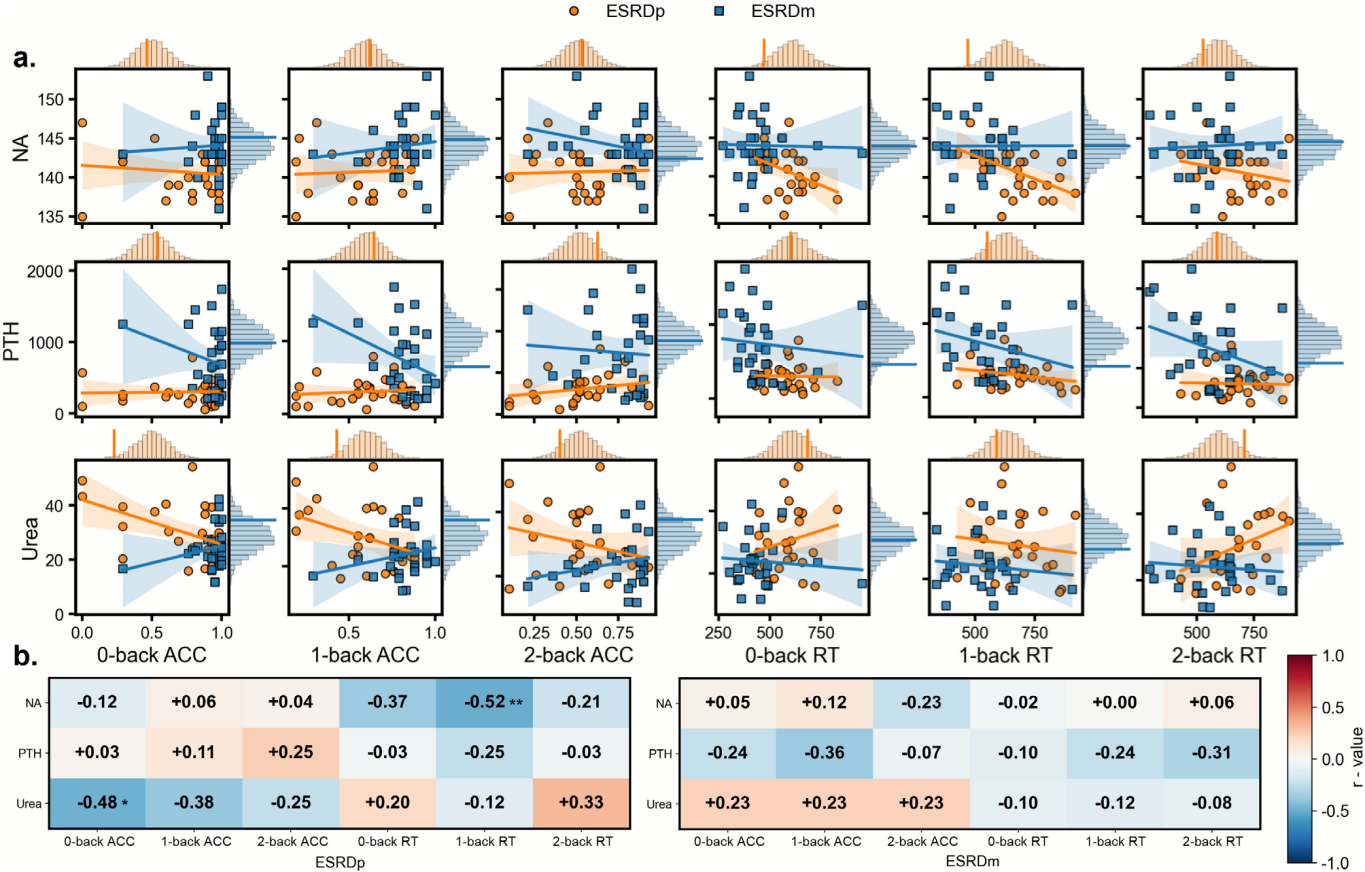
Associations between blood biochemistry and working‐memory performance in ESRD. (a) Scatterplots show correlations between blood markers that differed between groups (Na, PTH, and urea) and working‐memory performance (ACC and RT for 0‐, 1‐, and 2‐back). Orange circles and regression lines represent ESRDp, blue squares and lines represent ESRDm; shaded areas indicate 95% confidence intervals. Marginal histograms show the permutation‐based null distributions for each correlation within ESRDp (orange) and ESRDm (blue). (b) Heatmaps of correlation coefficients (*r*) for ESRDp (left) and ESRDm (right) for all marker–performance pairs. Asterisks indicate correlations that are significant based on permutation tests (*p_perm < 0.05, **p_perm < 0.01, uncorrected for FDR). ACC, accuracy; ESRDm, maintenance hemodialysis end‐stage renal disease patients; ESRDp, pre‐dialysis end‐stage renal disease patients; Na, sodium; PTH, parathyroid hormone; RT, reaction time.

We examined correlations between blood indices that showed significant between‐group differences and n‐back performance measures in ESRDp and ESRDm. As shown in Figure 3, in the ESRDp group, 0‐back ACC was negatively associated with urea (*r* = −0.481, *p* = 0.013, permutation *p* = 0.010) and 1‐back RT was negatively associated with serum sodium (*r* = −0.523, *p* = 0.009, permutation *p* = 0.010) at the uncorrected level; however, these associations did not remain significant after FDR correction. In the ESRDm group, no correlations between blood indices and working memory measures reached statistical significance.

### 3.4 Correlation Analysis Between SC/FC/SFC and Clinical Blood Indicators

**FIGURE 4:**
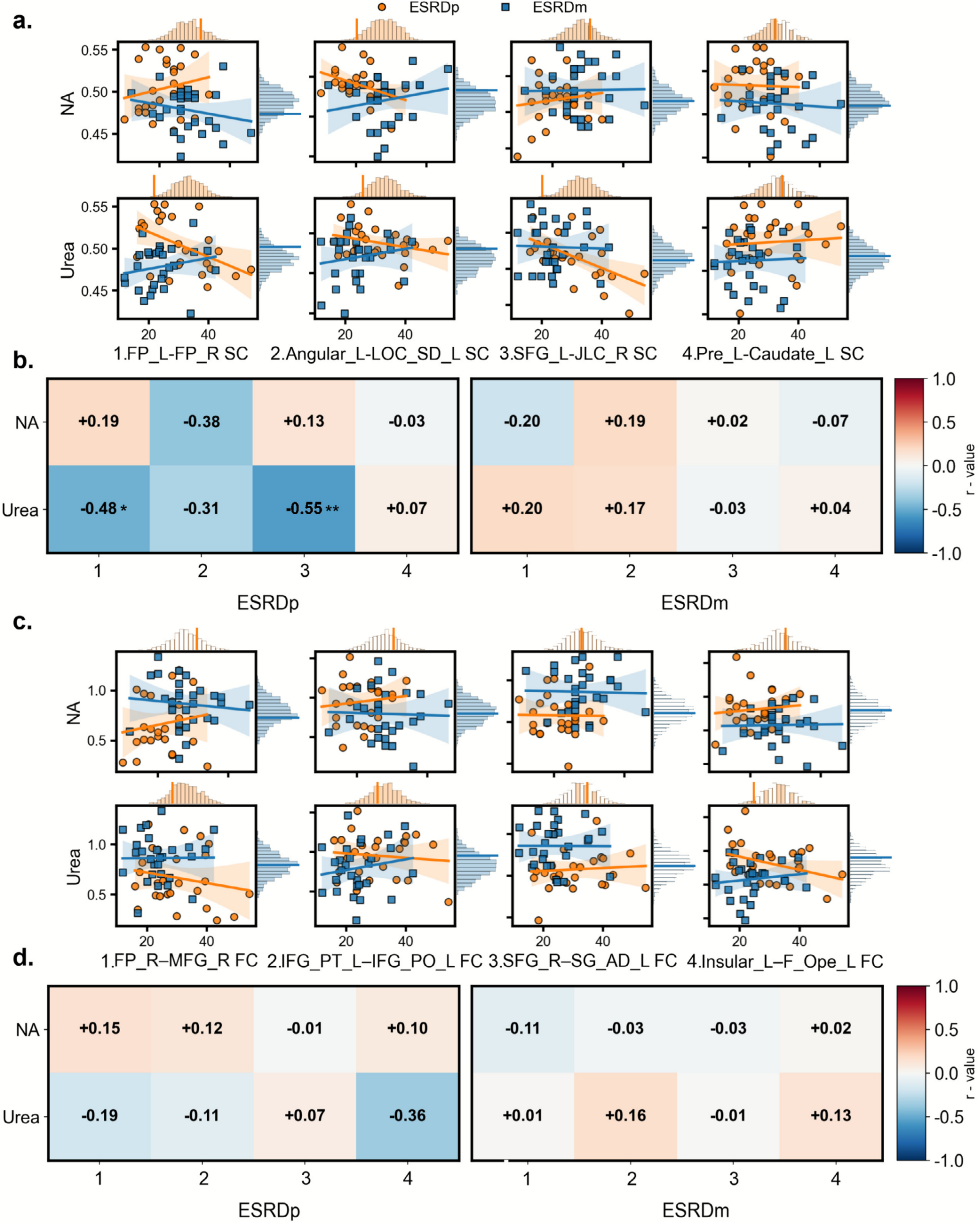
Correlations between blood biomarkers associated with n‐back performance and SC/FC in ESRD. (a) Scatterplots show the relationships between Na or urea and the SC strength of four edges (1–4) in ESRDp (orange circles and regression lines) and ESRDm (blue squares and lines); shaded bands indicate 95% confidence intervals. Marginal histograms depict the permutation‐based null distributions of the correlation coefficients for ESRDp (orange) and ESRDm (blue). (b) Heatmaps display correlation coefficients (*r*) between Na/urea and SC edges in ESRDp (left) and ESRDm (right). (c, d) Corresponding scatterplots and heatmaps for FC of four edges in ESRDp and ESRDm. Asterisks in the heatmaps denote correlations that are significant after multiple‐comparison correction and permutation testing (*p_FDR < 0.05, permutation *p* < 0.05; **p_FDR < 0.01, permutation *p* < 0.05). Angular_L, left angular gyrus; Caudate_L, left caudate; ESRDm, maintenance hemodialysis end‐stage renal disease patients; ESRDp, pre‐dialysis end‐stage renal disease patients; F_Ope_L, left frontal operculum cortex; FC, functional connectivity; FP_L, left frontal pole; FP_R, right frontal pole; IFG_PO_L, left inferior frontal gyrus, pars opercularis; IFG_PT_L, left inferior frontal gyrus, pars triangularis; Insular_L, left insular cortex; JLC_R, right juxtapositional lobule cortex (formerly supplementary motor cortex); Pre_L, left precentral gyrus; LOC_SD_L, left lateral occipital cortex, superior division; MFG_R, right middle frontal gyrus; Na, sodium; SC, structural connectivity; SFG_L, left superior frontal gyrus; SFG_R, right superior frontal gyrus; SG_AD_L, left supramarginal gyrus, anterior division.

We examined associations between blood indicators that were significantly related to n‐back performance and SC/FC/SFC measures. As shown in Figure 4, in ESRDp patients, higher urea levels were significantly associated with lower SC in FP_L–FP_R (*r* = −0.484, *p* = 0.012, *p*_FDR = 0.024, permutation *p* = 0.014) and SFG_L–JLC_R (*r* = −0.555, *p* = 0.003, *p*_FDR = 0.013, permutation *p* = 0.004). No significant correlations were observed between blood indices and FC in ESRDp.

**FIGURE 5:**
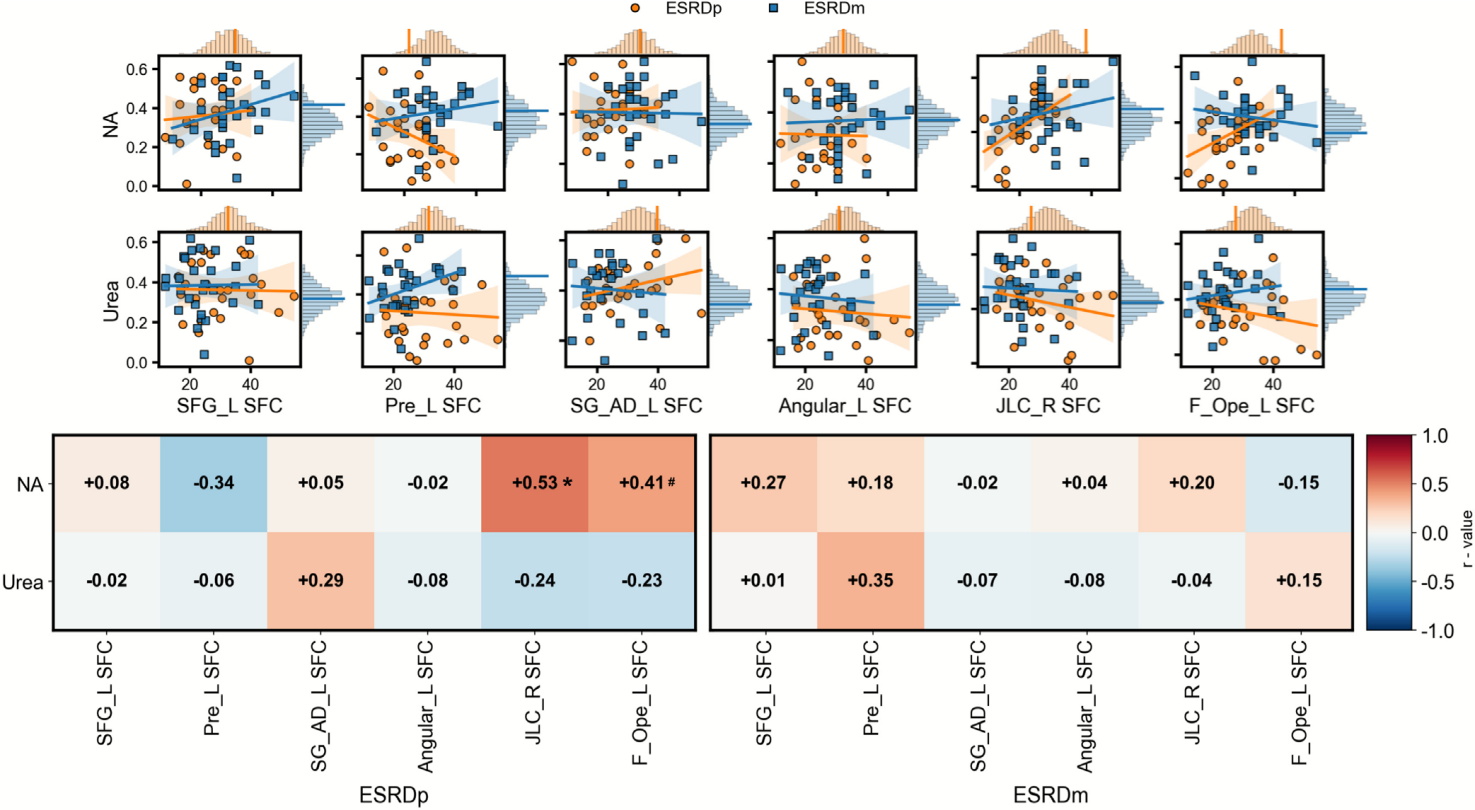
Correlations between blood biochemistries associated with SFC in ESRD. Scatterplots show relationships between Na or urea and SFC strength of six regions in ESRDp (orange circles and regression lines) and ESRDm (blue squares and lines); shaded bands indicate 95% confidence intervals. Marginal histograms show the permutation‐based null distributions for each correlation within ESRDp (orange) and ESRDm (blue). Heatmaps display correlation coefficients (*r*) between Na/urea and SFC in ESRDp (left) and ESRDm (right). # in the heatmaps denote correlations that are nominally significant (uncorrected *p* < 0.05). * in the heatmaps denote correlations that are significant in permutation tests after FDR correction (*p_FDR < 0.05, permutation *p* < 0.05). Angular_L, left angular gyrus; ESRDm, maintenance hemodialysis end‐stage renal disease patients; ESRDp, pre‐dialysis end‐stage renal disease patients; F_Ope_L, left frontal operculum cortex; JLC_R, right juxtapositional lobule cortex (formerly supplementary motor cortex); Na, sodium; Pre_L, left precentral gyrus; SFC, structural–functional coupling; SFG_L, left superior frontal gyrus; SG_AD_L, left supramarginal gyrus, anterior division.

As shown in Figure 5, in ESRDp patients, sodium levels were significantly positively correlated with SFC in JLC_R (*r* = 0.530, *p* = 0.005, *p*_FDR = 0.032, permutation *p* = 0.006). Sodium also showed a positive association with SFC in F_Ope_L (*r* = 0.413, *p* = 0.036, permutation *p* = 0.031); however, this latter association did not remain significant after FDR correction. No significant correlations between blood indicators and SC/FC/SFC measures were identified in ESRDm (Figures 4 and 5).

### 3.5 Correlation Analysis Between SC/FC/SFC and Working Memory Load

**FIGURE 6:**
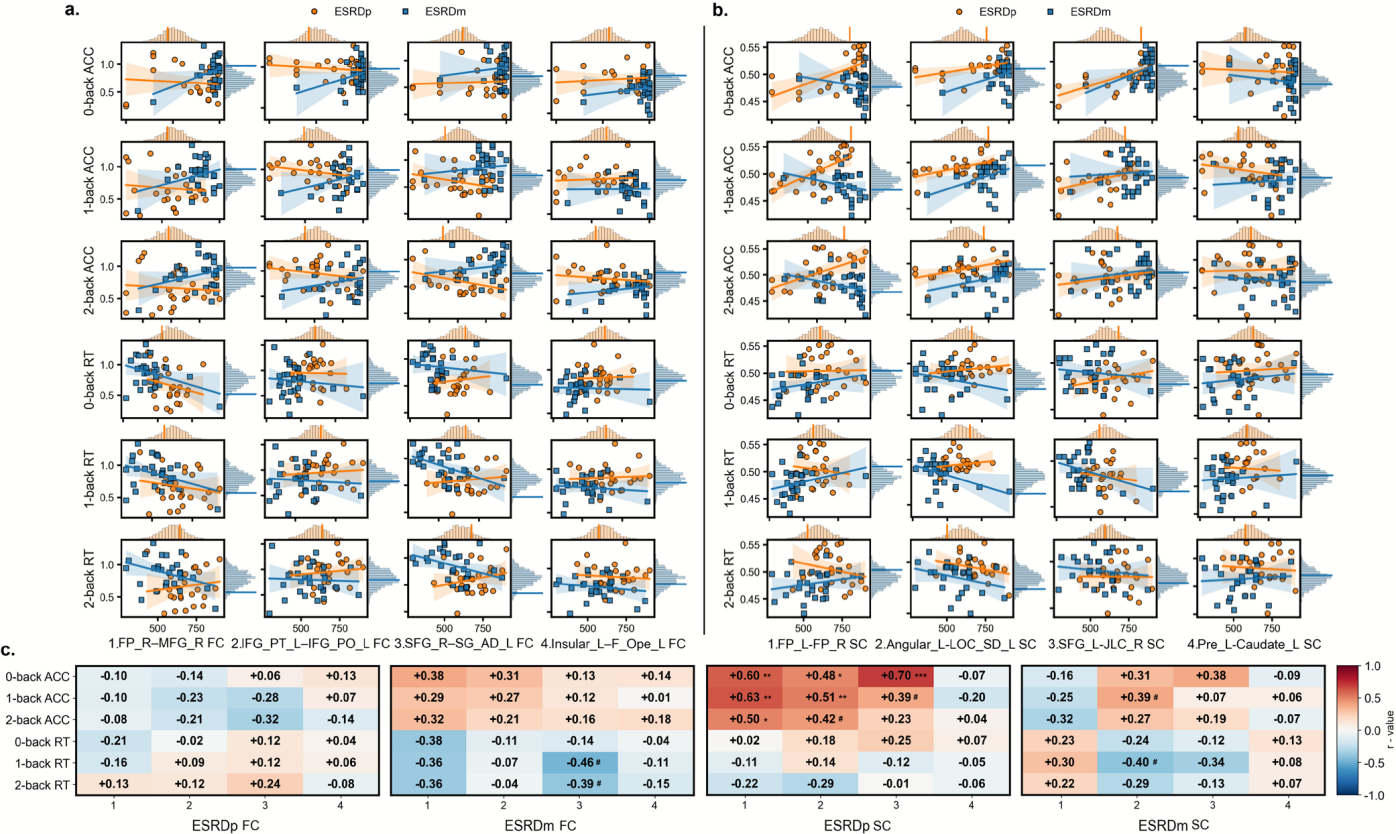
Correlations between n‐back performance and FC/SC in ESRD. (a) Scatterplots show relationships between n‐back performance (0‐, 1‐, and 2‐back ACC and RT) and FC of four edges (1–4) in ESRDp (orange circles and regression lines) and ESRDm (blue squares and lines); shaded bands indicate 95% confidence intervals. Marginal histograms depict the permutation‐based null distributions of the correlation coefficients for ESRDp (orange) and ESRDm (blue). (b) Scatterplots for SC illustrate relationships between n‐back performance and SC of four edges (1–4) in ESRDp and ESRDm, with the same color and plotting conventions as in (a). (c) Heatmaps summarize correlation coefficients (*r*) between n‐back measures and FC (left block) and SC (right block) for ESRDp and ESRDm. Asterisks (*) indicate correlations that remain significant after FDR correction and permutation testing (*p*_FDR < 0.05, permutation *p* < 0.05), whereas hash symbols (#) denote correlations that are nominally significant (uncorrected *p* < 0.05, permutation *p* < 0.05) but do not survive FDR correction. ACC, accuracy; Angular_L, left angular gyrus; Caudate_L, left caudate; ESRDm, maintenance hemodialysis end‐stage renal disease patients; ESRDp, pre‐dialysis end‐stage renal disease patients; F_Ope_L, left frontal operculum cortex; FC, functional connectivity; FP_L, left frontal pole; FP_R, right frontal pole; IFG_PO_L, left inferior frontal gyrus, pars opercularis; IFG_PT_L, left inferior frontal gyrus, pars triangularis; Insular_L, left insular cortex; JLC_R, right juxtapositional lobule cortex (formerly supplementary motor cortex); Pre_L, left precentral gyrus; LOC_SD_L, left lateral occipital cortex, superior division; MFG_R, right middle frontal gyrus; RT, reaction time; SC, structural connectivity; SFG_L, left superior frontal gyrus; SFG_R, right superior frontal gyrus; SG_AD_L, left supramarginal gyrus, anterior division.

As shown in Figure 6a,c, in ESRDm patients, FC in SFG_R–SG_AD_L was negatively correlated with 1‐back and 2‐back RT (1‐back: *r* = −0.464, *p* = 0.013, permutation *p* = 0.013; 2‐back: *r* = −0.388, *p* = 0.042, permutation *p* = 0.049). These correlations did not remain significant after FDR correction. No working memory measures were significantly correlated with FC in ESRDp.

As shown in Figure 6b,c, in ESRDm patients, 0‐back ACC correlated positively with SC in SFG_L–JLC_R (*r* = 0.383, *p* = 0.044, permutation *p* = 0.023), and 1‐back RT was negatively associated with SC in Angular_L–LOC_SD_L (*r* = −0.400, *p* = 0.032, permutation *p* = 0.040); these associations did not survive FDR correction. In the ESRDp group, 0‐back ACC correlated positively with SC in FP_L–FP_R (*r* = 0.602, *p* = 0.001, *p*_FDR = 0.003, permutation *p* = 0.003), Angular_L–LOC_SD_L (*r* = 0.480, *p* = 0.013, *p*_FDR = 0.039, permutation *p* = 0.020), and SFG_L–JLC_R (*r* = 0.696, *p* < 0.001, *p*_FDR < 0.001, permutation *p* < 0.001). Similarly, 1‐back ACC showed significant positive associations with SC in FP_L–FP_R (*r* = 0.631, *p* < 0.001, *p*_FDR = 0.003, permutation *p* = 0.002), Angular_L–LOC_SD_L (*r* = 0.510, *p* = 0.008, *p*_FDR = 0.039, permutation *p* = 0.008), and SFG_L–JLC_R (*r* = 0.386, *p* = 0.049, permutation *p* = 0.048; uncorrected). For 2‐back ACC, positive associations persisted with SC in FP_L–FP_R (*r* = 0.497, *p* = 0.010, *p*_FDR = 0.019, permutation *p* = 0.011) and Angular_L–LOC_SD_L (*r* = 0.424, *p* = 0.031, permutation *p* = 0.031; uncorrected).

**FIGURE 7:**
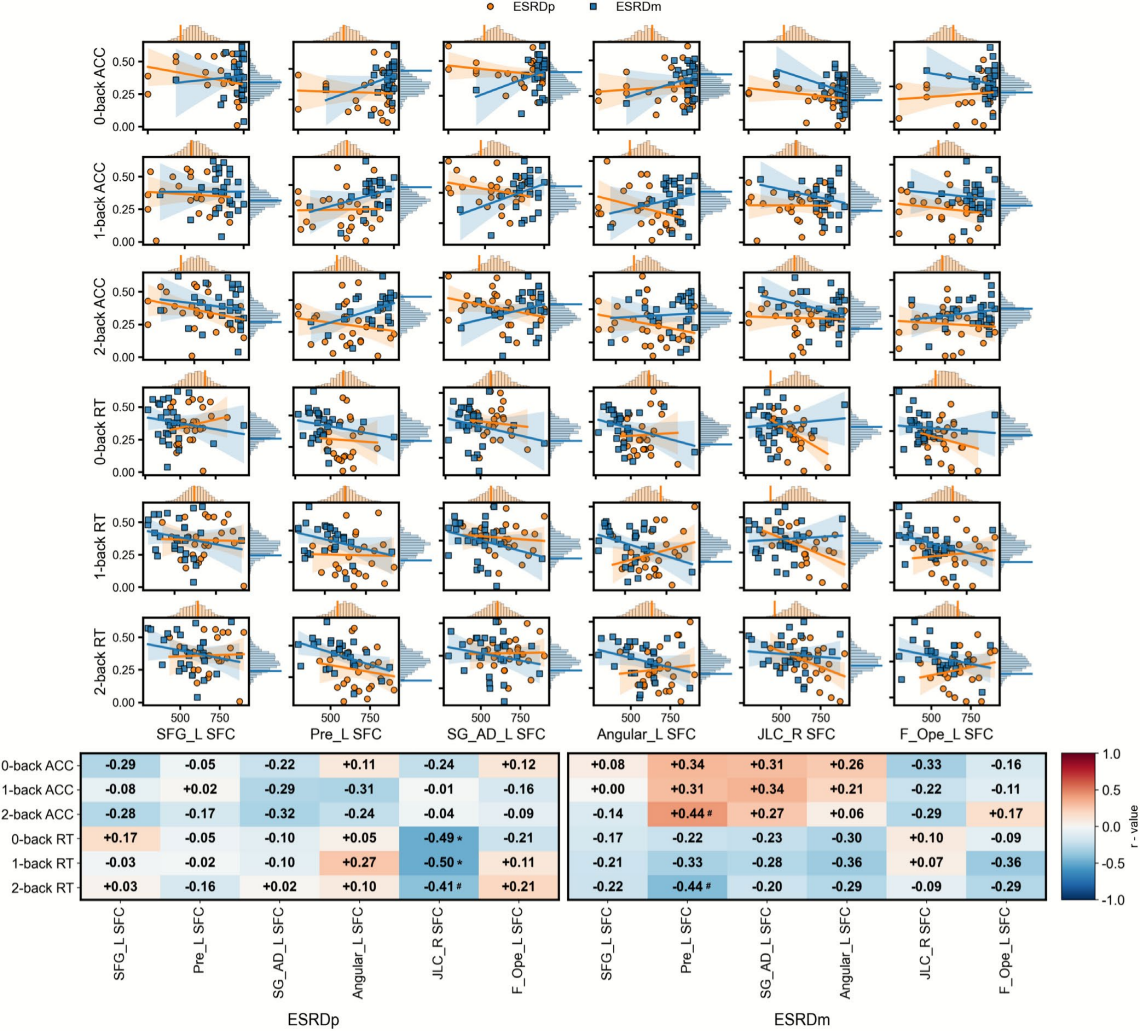
Correlations between n‐back performance and SFC in ESRD. Scatterplots show relationships between n‐back performance (0‐, 1‐, and 2‐back ACC and RT) and the SFC of six regions in ESRDp (orange circles and regression lines) and ESRDm (blue squares and lines); shaded bands indicate 95% confidence intervals. Marginal histograms depict the permutation‐based null distributions of the correlation coefficients for ESRDp (orange) and ESRDm (blue). Heatmaps summarize correlation coefficients (*r*) between n‐back measures and regional SFC in ESRDp (left block) and ESRDm (right block). In ESRDm, Asterisks (*) indicate correlations that remain significant after FDR correction (*p*_FDR < 0.05, permutation *p* < 0.05), whereas hash symbols (#) denote correlations that are nominally significant (uncorrected *p* < 0.05, permutation *p* < 0.05) but do not survive FDR correction. ACC, accuracy; Angular_L, left angular gyrus; ESRDm, maintenance hemodialysis end‐stage renal disease patients; ESRDp, pre‐dialysis end‐stage renal disease patients; F_Ope_L, left frontal operculum cortex; JLC_R, right juxtapositional lobule cortex (formerly supplementary motor cortex); Pre_L, left precentral gyrus; RT, reaction time; SFC, structural–functional coupling; SFG_L, left superior frontal gyrus; SG_AD_L, left supramarginal gyrus, anterior division.

As shown in Figure 7, in ESRDm patients, SFC in Pre_L was positively associated with 2‐back ACC (*r* = 0.437, *p* = 0.020, permutation *p* = 0.019) and negatively associated with 2‐back RT (*r* = −0.436, *p* = 0.020, permutation *p* = 0.024). However, these correlations did not survive FDR correction. In ESRDp patients, SFC in JLC_R was negatively correlated with RT across all loads (0‐back RT: *r* = −0.494, *p* = 0.010, *p*_FDR = 0.031, permutation *p* = 0.007; 1‐back RT: *r* = −0.496, *p* = 0.010, *p*_FDR = 0.031, permutation *p* = 0.012; 2‐back RT: *r* = −0.413, *p* = 0.036, permutation *p* = 0.032; uncorrected).

### 3.6 Mediation Analysis of SC/FC/SFC on the Relationships Between Blood Indicators and Working Memory Load

We tested whether *SC/FC/SFC* mediated the associations between altered blood biochemistry and working memory performance in ESRDp (Table 3; Figure S3), adjusting for hemoglobin and PTH.

**TABLE 3:** Mediation models relating serum urea and sodium to working memory loads.

Effect type	Path (predictor → outcome)	*B*	95% CI for *B*	*p*	*β*
Panel (A) Urea → SC → 0‐back accuracy					
a paths (X → M)	Urea → SFG.L–JLC.R SC (M1)	−0.002	−0.003 to −0.000	0.015	−0.526
	Urea → FP_L–FR_R SC (M2)	−0.001	−0.002 to 0.000	0.047	−0.414
b paths (M → Y)	SFG.L–JLC.R SC (M1) → 0‐back ACC	4.966	1.469 to 8.462	0.008	0.510
	FP_L–FR_R SC (M2) → 0‐back ACC	3.628	0.137 to 7.118	0.042	0.379
Total effect (*c*)	Urea → 0‐back ACC (no mediators)	−0.017	−0.030 to −0.005	0.010	−0.582
Direct effect (*c*')	Urea → 0‐back ACC|M1, M2	−0.005	−0.016 to 0.007	0.396	−0.156
Indirect effects (bootstrap)	Total indirect: Urea → (M1 + M2) → 0‐back ACC	−0.013	−0.021 to −0.002	—	−0.425
	Specific via SFG.L–JLC.R SC (M1)	−0.008	−0.014 to −0.001	—	−0.268
	Specific via FP_L–FR_R SC (M2)	−0.005	−0.011 to −0.001	—	−0.157
Panel (B) sodium → F_Ope_L SFC → 1‐back reaction time					
a path (X → M)	Sodium → F_Ope_L SFC (M)	0.018	0.005 to 0.032	0.010	0.570
b path (M → Y)	F_Ope_L SFC (M) → 1‐back RT	506.028	40.503 to 971.552	0.034	0.429
Total effect (*c*)	Sodium → 1‐back RT (no mediator)	−12.870	−29.265 to 3.525	0.119	−0.339
Direct effect (*c*')	Sodium → 1‐back RT|M	−22.141	−39.610 to −4.671	0.015	−0.584
Indirect effect (bootstrap)	Sodium → F_Ope_L SFC → 1‐back RT	9.270	1.679 to 20.504	—	0.244

*Note:* Panel A shows the parallel mediation model with serum urea as the predictor (X), SFG.L–JLC.R SC and FP_L–FR_R SC as mediators (M1, M2), and 0‐back accuracy as the outcome (Y). Panel B shows the mediation model with serum sodium as the predictor (X), F_Ope_L SFC as the mediator (M), and 1‐back reaction time as the outcome (Y). All models are adjusted for hemoglobin and PTH. *B* = unstandardized coefficient; *β* = standardized coefficient; *c* = total effect; *c*' = direct effect; ab = indirect effect (bootstrap, 5000 resamples).

Abbreviations: ACC, accuracy; Hb, hemoglobin; PTH, parathyroid hormone; RT, reaction time; SC, structural connectivity; SFC, structural‐functional coupling.

For urea, the parallel multiple mediation model showed that higher serum urea was associated with reduced SC in both SFG_L–JLC_R and FP_L–FP_R. In turn, higher SC in these two connections was related to better 0‐back ACC, and the direct effect of urea on 0‐back ACC became non‐significant after accounting for SC. Bootstrap analysis (5000 resamples) indicated a significant total indirect effect of urea on 0‐back ACC via SFG_L–JLC_R SC and FP_L–FP_R SC combined, consistent with a full mediation pattern whereby elevated urea impairs 0‐back performance primarily through disrupted structural connectivity.

For sodium, a separate mediation model revealed that lower serum sodium was associated with lower SFC in F_Ope_L, which in turn predicted longer 1‐back RT. The indirect effect of sodium on 1‐back RT via F_Ope_L SFC was significant, while the total effect of sodium was not. After including the mediator, the direct effect of sodium on 1‐back RT became significantly negative, suggesting an inconsistent (suppressor‐type) mediation pattern. Full parameter estimates for both mediation models are reported in Table 3.

## 4 Discussion

In our study, we aimed to characterize how WM‐related SC, FC, and SFC differ between ESRDp, ESRDm, and HCs, and to determine how these alterations relate to routine biochemical indices and WM performance. We found that ESRDp showed pronounced WM deficits and widespread disruption of frontoparietal SC and SFC, whereas ESRDm exhibited near‐normal WM performance and partially normalized network organization. Within ESRD, urea‐ and sodium‐related variations in frontoparietal SC and regional SFC statistically accounted for part of their associations with n‐back ACC and RT, even after adjusting for Hb and PTH. These findings provide a network‐level perspective on WM impairment in ESRD and highlight WM‐related SC and SFC, together with routinely collected biochemical markers, as promising candidate indicators of cognitive vulnerability.

### 4.1 Network Disruption and WM Impairment in Pre‐Dialysis ESRD

The first major observation is that ESRDp patients displayed marked deficits in n‐back performance, with reduced ACC and prolonged RT across 0‐, 1‐, and 2‐back conditions relative to both ESRDm and HCs. These findings align with epidemiological data indicating that up to half of patients with advanced CKD or ESRD meet criteria for mild cognitive impairment, with WM and executive dysfunction as prominent features [6, 33, 34]. Within the WM‐related networks, ESRDp showed reduced SC in callosal and fronto‐parietal association pathways (FP_L–FP_R, Angular_L–LOC_SD_L, SFG_L–JLC_R, Pre_L–Caudate_L) and decreased regional SFC in multiple frontal and parietal ROIs, together with selective FC alterations in dorsolateral prefrontal and supramarginal nodes. These convergent abnormalities suggest that ESRDp is characterized by both disruption of the structural backbone supporting WM networks and a local uncoupling between structural and functional organization. This pattern extends prior diffusion and resting‐state fMRI studies showing reduced frontoparietal SC and FC, cortical thinning, and disturbed intra‐ and inter‐hemispheric connectivity in ESRD, particularly in regions supporting attention and executive control [35, 36, 37, 38]. Notably, the most affected connections in our cohort are long‐range, highly myelinated association fibers linking dorsolateral prefrontal, premotor, and inferior parietal cortices—core components of the frontoparietal control network, which is metabolically demanding and situated in vascular “border‐zone” territories. These properties may render frontoparietal WM circuits particularly vulnerable to the combined effects of uremic toxins, microvascular dysfunction, and chronic metabolic stress in ESRD. The positive associations between SC strength (FP_L–FP_R, Angular_L–LOC_SD_L, SFG_L–JLC_R) and n‐back ACC across loads in ESRDp further support the functional relevance of these pathways for WM maintenance and updating. In line with connectomics work in healthy adults, stronger prefrontal–parietal SC and tighter structure–function alignment tend to predict better cognitive performance, particularly on tasks tapping goal‐directed control and WM, underscoring the importance of these long‐range association systems for cognitive resilience [23, 39].

### 4.2 Maintenance Hemodialysis and Partial Normalization of WM Networks

A second key finding is that ESRDm patients, who had been on stable hemodialysis, showed WM performance that was statistically indistinguishable from HCs, despite a history of advanced kidney failure. In our study, SC and SFC alterations were markedly less extensive in ESRDm than in ESRDp: several frontoparietal SC (e.g., FP_L–FP_R, Pre_L–Caudate_L) and regional SFC measures (e.g., Pre_L, LOC_SD_L) were significantly higher in ESRDm than in ESRDp and approached, though did not always reach, HC levels. These observations are consistent with prior longitudinal and cross‐sectional reports showing that initiation or maintenance of hemodialysis is associated with stabilization or partial improvement of global cognition and functional brain organization in some patients, although not uniformly so [37, 40, 41]. Importantly, residual SC and SFC deficits persisted in ESRDm, particularly in parietal association regions (e.g., Angular_L–LOC_SD_L SC, Post_L and F_Ope_L SFC), indicating that network restoration is incomplete. This aligns with structural MRI studies showing enduring cortical thinning and white‐matter microstructural damage in long‐term hemodialysis patients, even in those with relatively preserved global cognition [35, 36]. Overall, these findings suggest that maintenance hemodialysis may be associated with partial normalization or stabilization of WM‐related networks and behavior, while chronic brain injury and maladaptive plasticity remain and continue to constrain full recovery.

### 4.3 Biochemical Disturbances, Network Integrity, and WM Performance

By integrating routine laboratory measures with connectivity metrics, the present study provides evidence that specific biochemical disturbances are selectively linked to WM‐related network disruption. After FDR correction, ESRDp patients showed higher serum urea and lower sodium than ESRDm, with trends toward lower calcium and hemoglobin; PTH levels were markedly lower in ESRDp, reflecting differences in dialysis state and mineral metabolism. Among these indices, only urea and sodium showed robust correlations with connectivity measures and WM performance that survived multiple‐comparison control. In ESRDp, higher urea was associated with weaker SC in FP_L–FP_R and SFG_L–JLC_R, and these reduced SC strengths were, in turn, associated with poorer 0‐back accuracy. Mediation analyses adjusting for hemoglobin and PTH suggested that the statistical association between urea and WM accuracy was largely accounted for by SC in these two frontoparietal pathways, whereas the direct path from urea to behavior was not significant. These results fit with the broader concept of a “kidney–brain axis” in which uremic retention solutes and metabolic derangements contribute to blood–brain barrier dysfunction, neuroinflammation, and microvascular injury, ultimately disturbing cortical and subcortical networks critical for cognition [6, 14, 42, 43, 44]. Although urea itself is not considered the most neurotoxic solute, it is tightly linked to the overall uremic milieu and dialysis adequacy, and correlates with several middle‐molecule and protein‐bound toxins that exert direct neurotoxic and vascular effects [6]. In this context, the observed urea–SC–WM pathway in ESRDp likely reflects the aggregate impact of multiple uremic factors on long‐range association fibers supporting attention and response selection, rather than a specific effect of urea alone.

Sodium showed a different pattern: lower sodium in ESRDp was associated with longer 1‐back reaction times and with altered SFC, particularly in F_Ope_L and JLC_R, with SFC in F_Ope_L partially mediating the sodium–RT association. Rather than conceptualizing sodium as a uremic toxin, a more plausible interpretation is that lower sodium in this cohort indexes chronic fluid and osmotic imbalance, nutritional status, and dialysis‐independent homeostatic stress, all of which have been implicated in cognitive risk [45, 46]. Mild reductions in serum sodium, even within the clinically “normal” range, have been associated with slowed processing speed and attentional lapses, potentially via subtle cerebral edema and impaired neurotransmission [47]. In the present data, sodium‐related differences were restricted to ESRDp and did not explain WM performance in ESRDm, suggesting that these associations may be most pronounced in the pre‐dialysis milieu, when electrolyte and volume fluctuations are not yet managed by regular renal replacement therapy.

It is notable that other laboratory indices, including calcium, phosphate, cystatin C, and even hemoglobin and PTH, did not show robust mediating effects on WM measures despite group differences. This does not imply that anemia or secondary hyperparathyroidism is unimportant for brain health: both have been linked to cerebrovascular disease, white‐matter damage, and cognitive decline in CKD [6, 43]. Rather, in this WM‐focused paradigm and sample size, urea‐related SC changes and sodium‐related SFC alterations emerged as the most proximal network correlates of n‐back performance after adjusting for these covariates. These findings suggest that routinely monitored markers such as urea and sodium may carry information about underlying WM‐network integrity in ESRDp, and that integrating such biochemical indices with multimodal connectivity measures could help identify patients at heightened risk for WM disruption.

### 4.4 Structural–Functional Coupling as an Integrative Marker of WM Network Vulnerability

Beyond separate SC and FC measures, the regional SFC index used here captures the alignment between the presence and strength of white‐matter connections and the corresponding functional coupling profile for each node. ESRDp showed widespread reductions in SFC across frontal, parietal, and temporal WM nodes, suggesting a decoupling between structural scaffolding and spontaneous functional communication within WM networks. Such decoupling has been reported in several neurological and psychiatric conditions and has been linked to lower general cognitive ability and reduced network efficiency [23, 39, 48].

Our multimodal design also highlights that SC, FC, and SFC are not interchangeable but instead capture partially dissociable aspects of network vulnerability in ESRD. SC derived from diffusion tractography reflects relatively slow‐evolving microstructural injury—such as axonal loss, demyelination, and microvascular damage—and therefore indexes the cumulative impact of chronic kidney failure on the structural backbone of long‐range association fibers. In contrast, FC derived from resting‐state BOLD signals is more state‐dependent and can be shaped by both injury and compensatory reorganization, which may explain why FC alterations in our cohort were more regionally restricted and sometimes showed relative increases in ESRDm. SFC integrates these two levels by quantifying how closely a node's functional coupling profile adheres to its structural embedding, making it particularly sensitive to situations in which structure is only partially compromised but functional dynamics become inefficient or misaligned.

In the present study, reduced SFC in JLC_R was strongly associated with prolonged reaction times across all n‐back loads in ESRDp, even after FDR correction, while SFC in Pre_L was related to 2‐back performance in ESRDm. These patterns support the idea that SFC may be particularly sensitive to subtle network reorganization under chronic metabolic stress, capturing situations in which the underlying structural connections remain partially intact but functional dynamics become either “over‐flexible” (engaging atypical pathways) or “under‐responsive” (failing to recruit structurally available links). Recent work in healthy and clinical populations similarly indicates that regional SFC in frontoparietal systems is a robust correlate of individual differences in reasoning and WM, and that task‐related changes in SFC track the recruitment of distinct communication strategies under cognitive load: [23, 39].

Within the ESRD context, SFC may therefore provide a complementary biomarker to traditional SC and FC indices, reflecting both structural injury and compensatory functional reweighting. Multimodal metrics such as SC, SFC, and task‐relevant FC within WM networks may offer added value beyond global cognitive screening, particularly for identifying ESRD patients who remain at high risk of cognitive decline despite standard dialysis adequacy targets and for monitoring the effects of interventions aimed at preserving brain health.

### 4.5 Limitations

This study has several important limitations. First, its cross‐sectional design prevents causal inference. Although group differences between ESRDp and ESRDm are compatible with partial normalization of WM networks under maintenance hemodialysis, they do not prove that dialysis itself causes network recovery; longitudinal imaging starting before dialysis initiation will be needed to clarify temporal relationships between network change and clinical improvement. Second, we relied on routine laboratory indices and did not measure middle‐molecule or protein‐bound uremic toxins (e.g., indoxyl sulfate, p‐cresyl sulfate), which are increasingly implicated in neurovascular and cognitive injury in CKD [9, 42]; future work should include broader toxin panels and markers of inflammation, oxidative stress, and dialysis dose to disentangle their specific contributions. Third, the modest sample sizes limited statistical power for detailed subgroup/stratified analyses (e.g., by ESRD etiology, comorbidity profiles, or anemia/mineral‐bone disorder markers such as hemoglobin and PTH). To mitigate potential confounding, key clinical variables were carefully characterized and incorporated into the statistical models, and the distribution of major biopsy‐proven primary glomerular diseases was broadly comparable between ESRDp and ESRDm. Nevertheless, small numbers within each etiologic subgroup may have reduced sensitivity to detect etiology‐specific patterns, and subtle subgroup effects on brain networks cannot be fully excluded. Therefore, larger multicenter cohorts with longitudinal follow‐up will be important to replicate these findings and to further evaluate sources of heterogeneity, thereby strengthening generalizability. Fourth, comorbidities and medications (e.g., erythropoietin, antihypertensives) were not fully modeled, and more systematic characterization of treatment regimens and nutritional status will be important in future studies.

## 5 Conclusion

Our study shows that WM–related SC, FC, and particularly SFC are systematically altered across the ESRDp and ESRDm groups. ESRDp patients exhibited pronounced disruption and decoupling of frontoparietal WM networks together with impaired n‐back performance, whereas patients on ESRDm showed near‐normal WM behavior and partially normalized, but still incomplete, network organization. Within ESRD, individual differences in urea‐ and sodium‐related SC and SFC were closely associated with variation in WM performance, supporting the concept of a kidney–brain axis in which systemic biochemical disturbances are linked to frontoparietal network vulnerability. Our findings highlight WM‐related SC and SFC, in combination with routinely collected biochemical markers, as promising candidate indicators of cognitive risk in ESRD that warrant confirmation and further development in larger, longitudinal, and interventional studies.

## Author Contributions

Xiaoling Xu, Junya Mu, Shaohui Ma and Siyao Liu: methodology, data curation, software, and writing (original draft). Zhaoyao Luo, Wen Gu, Huijie Yuan: data curation, visualization, and investigation. Qiange Zhu, Xinyi Zhu, and Jianjun Zhang: conceptualization, methodology, software, formal analysis. Ming Zhang and Peng Li: funding acquisition, writing (review and editing). Junya Mu: funding acquisition, conceptualization, supervision, and writing (review and editing). All authors contributed to the article and approved the submitted version.

## Funding

This work was supported by the Health Research and Innovation Capacity Strengthening Platform Program of Shaanxi Province (Grant Number 2023PT‐09), the Clinical Research Award of the First Affiliated Hospital of Xi'an Jiaotong University (Grant Number XJTU1AF‐CRF‐2023–021), the Key Research and Development Plan of Xianyang City (Grant No. L2023‐ZDYF‐SF‐035 and L2022ZDYF‐SF‐026), and the Science and Technology Research Project of Shaanxi Nuclear Industry Group Co. Ltd. (Grant No. 61240302).

## Ethics Statement

Ethical approval for this study was granted by the Institutional Review Board of the First Affiliated Hospital, School of Medicine, Xi'an Jiaotong University (approval ID: XJTU1AF‐CRF‐2018‐006). All procedures complied with the principles of the Declaration of Helsinki. Before enrolment, every participant received a detailed description of the study and provided written informed consent.

## Consent

The authors have nothing to report.

## Conflicts of Interest

The authors declare no conflicts of interest.

## Supporting information


**Table S1:** WM related nodes with anatomical labels, abbreviations, and MNI coordinates.
**Table S2:** Group differences among HCs, ESRDp, and ESRDm in SFC.
**Figure S1:** Group differences among ESRDp, ESRDm, and HCs in WM load.
**Figure S2:** Group differences between ESRDp and ESRDm in blood biochemistry variables.
**Figure S3:** Mediation analysis results in the ESRDp group.
**Data S1:** Shapiro–Wilk normality tests for n‐back performance metrics and blood biochemistry variables in ESRDp and ESRDm.

## Data Availability

The data that support the findings of this study are available from the corresponding author upon reasonable request.
