# Supplementary material for: Structural Connectivity Disruption and Structural–Functional Decoupling in Working Memory Networks Across Pre‐Dialysis and Maintenance Hemodialysis End‐Stage Renal Disease Patients
Source: CNS Neurosci Ther. 2026 Jan 19;32(1):e70761. doi: 10.1002/cns.70761 (PMC12813873; doi:10.1002/cns.70761)
Supplement: Supplementary file 1 — Table S1: WM related nodes with anatomical labels, abbreviations, and MNI coordinates. Table S2: Group differences among HCs, ESRDp, and ESRDm in SFC. Figure S1: Group differences among ESRDp, ESRDm, and HCs in WM load. Figure S2: Group differences between ESRDp and ESRDm in blood biochemistry variables. Figure S3: Mediation analysis results in the ESRDp group. Data S1: Shapiro–Wilk normality tests for n‐back performance metrics and blood biochemistry variables in ESRDp and ESRDm. [file CNS-32-e70761-s001.docx]

**Results**

*Normality of n-back performance metrics*

Shapiro–Wilk tests were applied to assess the distribution of ACC and RT data for each group and WM load level. In HCs, 0-back ACC (p < 0.001), 1-backACC (p =0.015), and 2-back ACC (p = 0.043) deviated from normality, whereas RT at 0-back (p = 0.151), 1-back (p = 0.011), and 2-back (p = 0.122) were normally distributed. In the ESRD_p_ group, only 0-back ACC was non-normal (p < 0.001); 1-back ACC (p = 0.100), 2-back ACC (p = 0.480), and RT at all loads (0-back, p = 0.362; 1-back, p = 0.974; 2-back, p = 0.998) met normality criteria. For ESRD_m_, ACC at 0-back (p < 0.001), 1-back (p < 0.001), and 2-back (p = 0.001), as well as RT at 0-back (p < 0.001) and 1-back (p = 0.047), were non-normal; only 2-back RT conformed to normality (p = 0.682).

*Normality of blood biochemistry tests*

In ESRD_p_ group, serum calcium was the sole variable to violate normality (p = 0.029), while hemoglobin (p = 0.341), potassium (p = 0.741), sodium (p = 0.686), phosphate (p = 0.847), parathyroid hormone (p = 0.073), creatinine (p = 0.175), urea (p = 0.218), and cystatin C (p = 0.815) were normally distributed. In ESRD_m_ group, hemoglobin (p = 0.007), calcium (p = 0.026), and cystatin C (p = 0.038) were non-normal, whereas potassium (p = 0.214), sodium (p = 0.439), phosphate (p = 0.117), parathyroid hormone (p = 0.069), creatinine (p = 0.096), and urea (p = 0.137) satisfied normality.

| **Table S1 working memory-related nodes** | | | | |
| --- | --- | --- | --- | --- |
| **Anatomical labels** | **Abbreviation** | **MNI coordinates** | | |
|  |  | **x** | **y** | **z** |
| Left Frontal Pole | FP_L | -25 | 53 | 8 |
| Right Frontal Pole | FP_R | 26 | 52 | 8 |
| Left Insular Cortex | Insular_L | -37 | 1 | 0 |
| Right Insular Cortex | Insula.R | 38 | 3 | 0 |
| Left Superior Frontal Gyrus | SFG_L | -14 | 19 | 57 |
| Right Superior Frontal Gyrus | SFG_R | 15 | 19 | 57 |
| Left Middle Frontal Gyrus | MFG_L | -38 | 19 | 42 |
| Right Middle Frontal Gyrus | MFG_R | 39 | 19 | 43 |
| Left Inferior Frontal Gyrus, pars triangularis | IFG_PT_L | -50 | 29 | 9 |
| Right Inferior Frontal Gyrus, pars triangularis | IFG_PT_R | 52 | 28 | 8 |
| Left Inferior Frontal Gyrus, pars opercularis | IFG_PO_L | -51 | 15 | 15 |
| Right Inferior Frontal Gyrus, pars opercularis | IFG_PO_R | 52 | 15 | 16 |
| Left Precentral Gyrus | Pre_L | -34 | -12 | 49 |
| Right Precentral Gyrus | Pre_R | 35 | -11 | 50 |
| Left Postcentral Gyrus | Post_L | -39 | -28 | 52 |
| Right Postcentral Gyrus | Post_R | 38 | -26 | 53 |
| Left Superior Parietal Lobule | SPL_L | -29 | -50 | 58 |
| Right Superior Parietal Lobule | SPL_R | 29 | -48 | 59 |
| Left Supramarginal Gyrus, anterior division | SG_AD_L | -57 | -33 | 37 |
| Right Supramarginal Gyrus, anterior division | SG_AD_R | 59 | -27 | 38 |
| Left Supramarginal Gyrus, posterior division | SG_PD_L | -55 | -46 | 33 |
| Right Supramarginal Gyrus, posterior division | SG_PD_R | 55 | -40 | 34 |
| Left Angular Gyrus | Angular_L | -50 | -56 | 30 |
| Right Angular Gyrus | Angular_R | 52 | -52 | 32 |
| Left Lateral Occipital Cortex, superior division | LOC_SD_L | -32 | -73 | 38 |
| Right Lateral Occipital Cortex, superior division | LOC_SD_R | 33 | -71 | 39 |
| Left Juxtapositional Lobule Cortex  (formerly Supplementary Motor Cortex) | JLC_L | -6 | -3 | 56 |
| Right Juxtapositional Lobule Cortex  (formerly Supplementary Motor Cortex) | JLC_R | 6 | -3 | 58 |
| Left Paracingulate Gyrus | PCG_L | -6 | 37 | 21 |
| Right Paracingulate Gyrus | PCG_R | 7 | 37 | 23 |
| Left Precuneous Cortex | PCE_L | -8 | -60 | 37 |
| Right Precuneous Cortex | PCE_R | 9 | -58 | 39 |
| Left Frontal Orbital Cortex | F_Orb_L | -30 | 24 | -16 |
| Right Frontal Orbital Cortex | F_Orb_R | 29 | 23 | -16 |
| Left Frontal Operculum Cortex | F_Ope_L | -40 | 18 | 5 |
| Right Frontal Operculum Cortex | F_Ope_R | 41 | 19 | 5 |
| Left Thalamus | Thalamus_L | -10 | -19 | 6 |
| Right Thalamus | Thalamus_R | 11 | -18 | 7 |
| Left Caudate | Caudate_L | -13 | 9 | 10 |
| Right Caudate | Caudate_R | 13 | 10 | 11 |

**Table S2. Results of group difference among HCs , ESRD_p_ and ESRD_m_ in SFC**

| **Regions** | **HCs**  **mean (SE)** | **ESRD_p_**  **mean (SE)** | **ESRD_m_**  **mean (SE)** | ***F*** | ***P_FDR*** | ***t_1_*** | ***P_1__FDR*** | ***t_2_*** | ***P_2__FDR*** | ***t_3_*** | ***P_3__FDR*** |
| --- | --- | --- | --- | --- | --- | --- | --- | --- | --- | --- | --- |
| Pre_L | 0.464 (0.018) | 0.305(0.030) | 0.423(0.023) | 12.678 | <0.001 | 4.523 | <0.001 | 1.450 | 0.193 | -3.130 | 0.011 |
| JLC_R | 0.533(0.020) | 0.428(0.019) | 0.474(0.019) | 7.174 | 0.002 | 3.863 | 0.002 | 2.166 | 0.068 | -1.758 | 0.133 |
| F_Ope_L | 0.481(0.017) | 0.356(0.019) | 0.420(0.016) | 12.570 | <0.001 | 4.930 | <0.001 | 2.631 | 0.027 | -2.642 | 0.027 |
| Angular_L | 0.413(0.016) | 0.313(0.024) | 0.368(0.021) | 6.545 | 0.003 | 3.441 | 0.006 | 1.694 | 0.135 | -1.745 | 0.133 |
| LOC_SD_L | 0.450(0.024) | 0.325(0.031) | 0.417(0.021) | 5.748 | 0.006 | 3.162 | 0.010 | 1.019 | 0.374 | -2.241 | 0.033 |
| Post_L | 0.491(0.015) | 0.342(0.028) | 0.419(0.023) | 13.686 | <0.001 | 4.774 | <0.001 | 2.685 | 0.030 | -4.381^d^ | <0.001 |

t1/ *P_1__FDR*: Statistical comparison between HCs and ESRD_p_ groups;
t2/ *P_2__FDR*: Statistical comparison between HCs and ESRD_m_ groups;
t3/ *P_3__FDR*: Statistical comparison between ESRD_p_ and ESRD_m_ groups.

Abbreviation: HCs: Healthy Controls; ESRD_p_: pre-dialysis ESRD patients; ESRD_m_: maintenance hemodialysis ESRD patients; ESRD: End-stage renal disease; SFC: structural–functional coupling; F_Ope_L: left frontal operculum cortex; JLC_R: right juxtapositional lobule cortex (formerly supplementary motor cortex); Pre_L: left precentral gyrus; Angular_L: left angular gyrus; LOC_SD_L: left lateral occipital cortex, superior division; Post_L: Left postcentral gyrus.


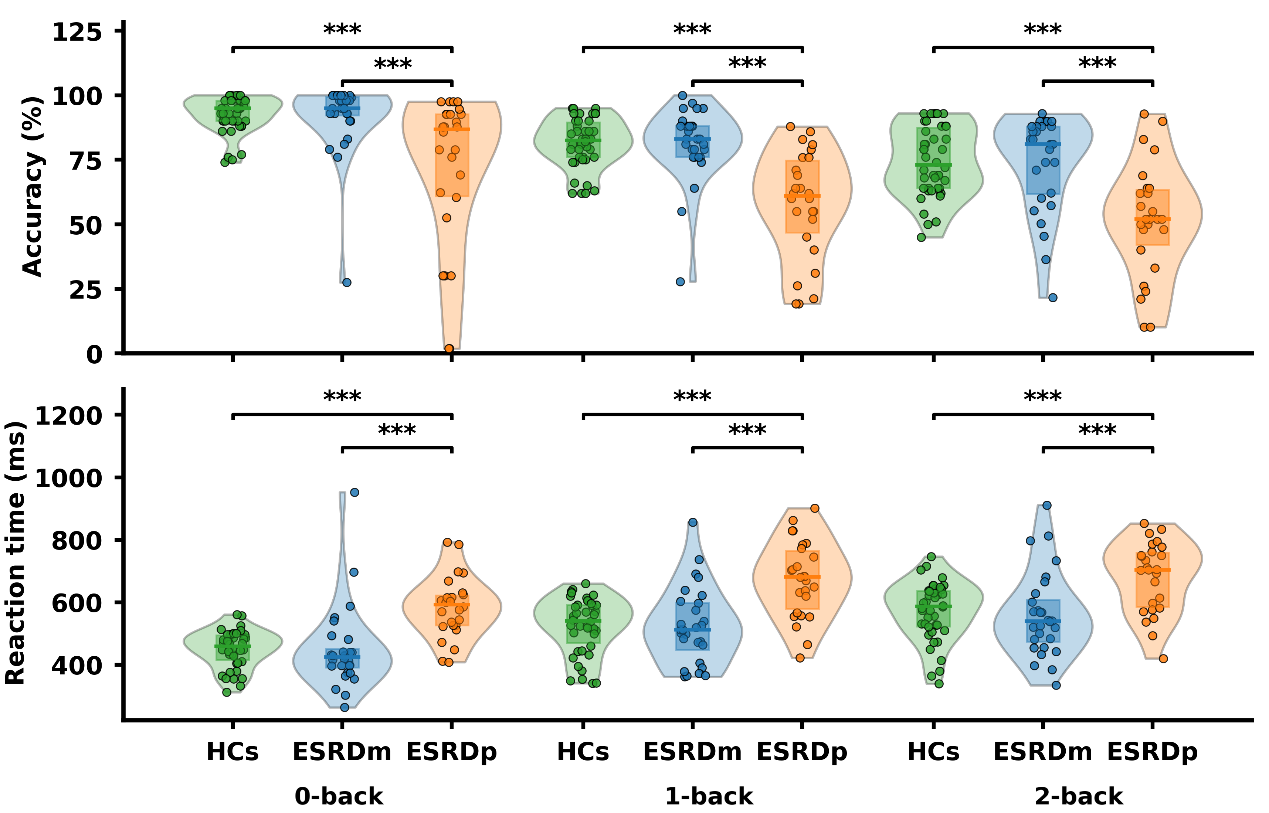


Figure S1. Group differences among ESRDp, ESRDm, and HCs in working memory load.

Abbreviation: ESRDp: pre-dialysis end-stage renal disease patients; ESRDm: maintenance hemodialysis end-stage renal disease patients; HCs: healthy controls.

*p < 0.05, **p < 0.01, ***p < 0.001.


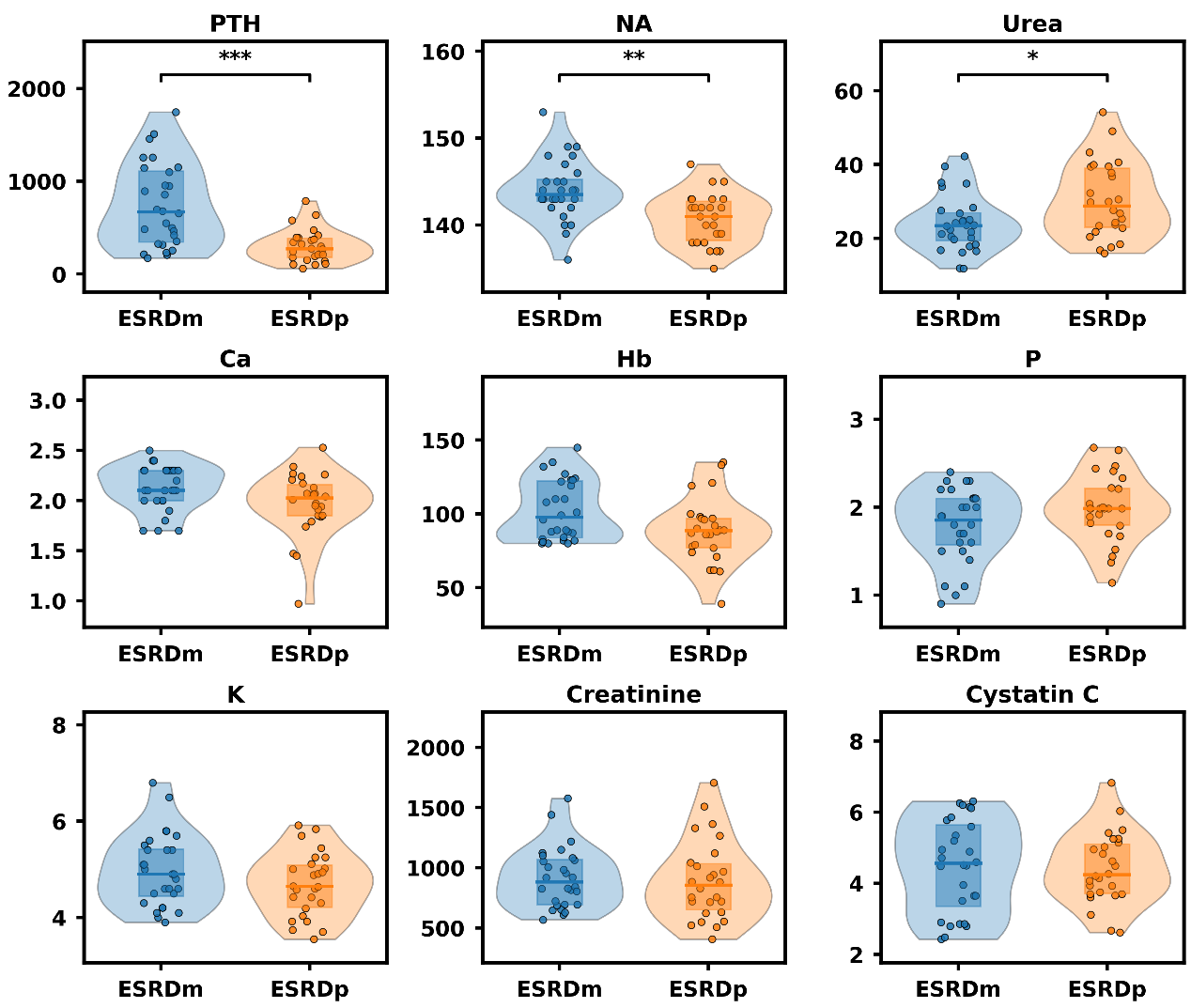


Figure S2. Group differences among ESRDp and ESRDm, in blood biomarkers.

Abbreviation: ESRDp: pre-dialysis end-stage renal disease patients; ESRDm: maintenance hemodialysis end-stage renal disease patients; HCs: healthy controls; PTH:Parathyroid Hormone; CA: Calcium; K:Potassium; NA:Sodium; P:Phosphorus; Hb:hemoglobin.

*p < 0.05, **p < 0.01, ***p < 0.001.


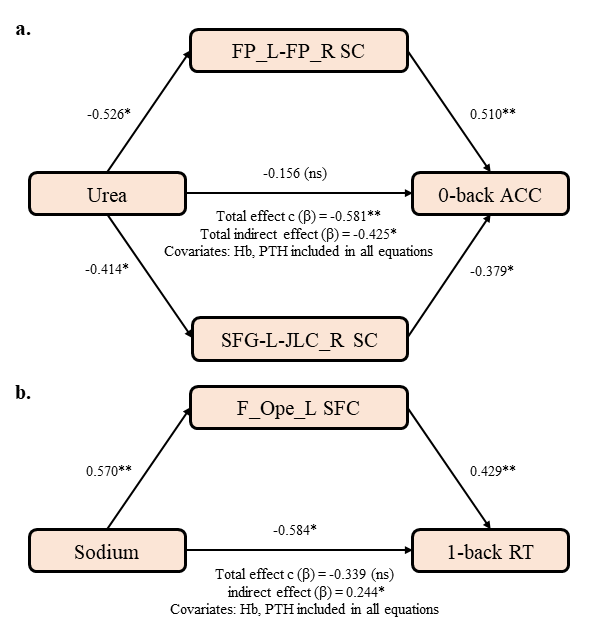


Figure S3. Mediation analysis results in the ESRDp group.

(a) Lower SC between FP_L–FP_R (M₁) and SFG_L–JLC_R (M₂) mediates the effect of urea (X) on 0-back ACC (Y). (b) Lower SFC in F_Ope_L mediates the effect of sodium (X) on 1-back RT (Y). All mediation effects are reported as standardized regression coefficients.

Abbreviation: ESRDp :pre-dialysis end-stage renal disease patients; ACC: accuracy; RT: reaction time; SC: structural connectivity; SFC: structural–functional coupling; FP_L: left frontal pole; FP_R: right frontal pole; SFG_L: left superior frontal gyrus; JLC_R: right juxtapositional lobule cortex (formerly supplementary motor cortex); F_Ope_L: left frontal operculum cortex.
